# Area PEc Neurons Use a Multiphasic Pattern of Activity to Signal the Spatial Properties of Optic Flow

**DOI:** 10.1155/2017/6495872

**Published:** 2017-11-19

**Authors:** Milena Raffi, Alessandro Piras, Roberta Calzavara, Salvatore Squatrito

**Affiliations:** Department of Biomedical and Neuromotor Sciences, University of Bologna, Bologna, Italy

## Abstract

The cortical representation of visual perception requires the integration of several-signal processing distributed across many cortical areas, but the neural substrates of such perception are largely unknown. The type of firing pattern exhibited by single neurons is an important indicator of dynamic circuitry within or across cortical areas. Neurons in area PEc are involved in the spatial mapping of the visual field; thus, we sought to analyze the firing pattern of activity of PEc optic flow neurons to shed some light on the cortical processing of visual signals. We quantified the firing activity of 152 optic flow neurons using a spline interpolation function, which allowed determining onset, end, and latency of each neuronal response. We found that many PEc neurons showed multiphasic activity, which is strictly related to the position of the eye and to the position of the focus of expansion (FOE) of the flow field. PEc neurons showed a multiphasic activity comprised of excitatory phases interspersed with inhibitory pauses. This phasic pattern seems to be a very efficient way to signal the spatial location of visual stimuli, given that the same neuron sends different firing patterns according to a specific combination of FOE/eye position.

## 1. Introduction

Area PEc is a multimodal area of the posterior parietal cortex involved in several functional processes, including optic flow processing [1–4], reaching arm movements [5, 6], and eye position encoding [7]. PEc neurons are directly connected to motor neurons in both premotor area F2 [8–13] and primary motor cortex [8]. Area PEc corresponds to the caudal part of area 5 according to the nomenclature of Pandya and Seltzer [14].

The processing of visual signals in the cerebral cortex involves various aspects of neuronal properties. Neurons can signal information using two coding strategies, the rate coding and the time coding [15]. In the rate coding, the firing rate of the neuron is crucial, while in the time coding the timing of individual spikes presents important information. The neural firing pattern is an important indicator of dynamic circuitry within a neuronal population. The “ramping activity” has been defined as a consistent increase or decrease in neuronal firing rate across behaviorally relevant epochs of time [16]. The so-called “ramping” is a common pattern of activity observed across cortical regions, such as prefrontal [16], cingulate [17], and frontal eye field [18]. Ding and Gold [18] found that the activity of many neurons in the frontal eye field depended on the monkey's saccadic choice and/or strength of the motion stimulus used to generate such choice. In premotor areas, movements typically start when the neural activity reaches a threshold, although it is still unclear how this threshold is determined [19]. Similar properties are shown by neurons in the posterior parietal cortex [6, 20]. Maimon and Assad [21] found that during the execution of arm movements, neurons in the parietal area 5 show a ramping-like activity as well as a burst/pause neural activity. These reaching-related neurons ramped up or down their firing rate prior to the beginning of movement, and such gradual changes in firing were not related to occurring differences in the visual input presentation.

The visual perception is an important aspect for generation of action in that relevant behaviors are often produced as response/reaction to visual stimuli, particularly in experimental context. Lee and Assad [22] and Maimon and Assad [21] hypothesized that the initiation of concerted movements would imply a systems-level threshold for movement initiation. Self-timed movements are potentially triggered by a network activity that reaches threshold gradually. The abrupt appearance of a sensory stimulus or cue signaling to produce the behavioral response may trigger the system to reach threshold more rapidly. According to this framework, when studying the corticocortical processing in action generation, it is critical to understand the role of visual stimuli in triggering movements.

When an observer moves through the environment, the visual perception is guaranteed by optic flow processing [23, 24]; such perception is crucial for action generation during self-motion. The aim of the present study was to analyze the firing pattern of the activity of PEc optic flow neurons, moving beyond the classical mean firing rate. We found that PEc neurons show a burst/pause neural activity, sometimes similar to the ramping-like activity described in reaching neurons in area 5 [6, 20, 21]. Area PEc might then represent an important node in the loop responsible for the amplification or reduction of neural responses through the processing of visual signal.

## 2. Methods

For this study, we reexamined the data of the experiments performed on five hemispheres of three male macaques. Monkeys* (M. fascicularis)* were bought from R.C. Hartelust B.V., P.O. Box  2170, 5001 CD Tilburg, The Netherlands. All experimental procedures were performed with the control of the veterinary staff of the University of Bologna, after receiving approval by the Committee on Ethics in Animal Experimentation of Bologna University and governmental approval, in compliance with the Italian guidelines for care and use of laboratory animals (Italian Legislative Decree 26/2014; in accordance with the European Community Council Directive 2010/63/UE on animal welfare). All efforts were made to minimize animal suffering.

### 2.1. Surgery

Surgical procedures are identical to those reported in previous papers [1–3, 7, 25]. Briefly, a metal recording chamber of 18 mm inner diameter was placed on the skull midline above the parietooccipital sulcus (POS). The chamber center was placed at stereotaxic coordinates. A metal head holder was also fixed on the bone by titanium screws and bone cement (Palacos®). All surgical procedures were performed under general anesthesia (Thiopental Sodium 15 mg/Kg i.v.). Analgesics and antibiotics were given for several days after surgery (Ketorolac Tromethamine, 30 mg/die i.m.; Benzathine Benzylpenicillin 0.1 ml/Kg i.m.) under control of the veterinary staff.

The extracellular recordings of single neurons were performed in both hemispheres of the first and the second monkeys (named ME and MF, resp.) and in the right hemisphere of the third monkey (named MG) using glass-coated elgiloy or tungsten microelectrodes passing through the dura mater. The extracellular recordings were performed on the dorsal surface of area PEc [14]. Data acquisition was made by a multi-spike-sorter system for electrophysiological recordings (Alpha-Omega Inc.).

### 2.2. Experimental Paradigm and Stimuli

The experimental paradigm and stimuli are identical to those described in previous papers [1, 2]. Briefly, the monkey was seated in a monkey chair in front of a dark screen of a 19′′ with a resolution of 1280 × 1024 pixels. The screen covered 60 × 70° of visual field and was placed 28.5 cm from the monkeys' eyes. The refresh rate of the monitor was 60 Hz. The visual responsiveness of each neuron was firstly evaluated moving a white luminous bar (3 cd/m) on the screen; this also allowed mapping the visual receptive field. Then, the neuronal activity was recorded with the optic flow paradigm described below. Optic flow stimuli were formed by 1000 randomly distributed white dots (3 cd/m^2^), which moved at an average perceived speed of about 10°/sec. The optic flow stimulus was full screen size.

**Figure 1:**
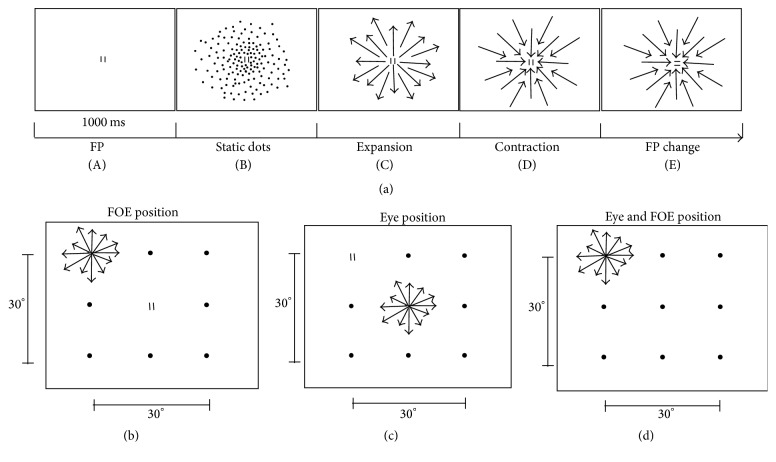
Stimuli. (a) Temporal sequence of the behavioral task. Monkeys were trained to push a lever when the FP appeared on the screen (A). After one second, 1000 random dots appeared (B). After one second the expansion stimulus started at a speed of about 10°/s (C), followed after one second by the contraction stimulus (D). The monkey had to detect a change in FP orientation (E) and release the lever within a maximum reaction time of 500 ms. (b) Retinotopic condition. The FP was presented in the center of the screen, and FOE in one of nine locations in a 3 × 3 grid at 15° distance each. Black circles indicate other FOE peripheral positions. (c) Eye position test. The FOE was presented in the center of the screen, while FP was in one of the nine peripheral locations. Black circles indicate other FP peripheral positions. (d) Angle of gaze condition. FP and FOE were displayed concentrically in one of the nine locations. Black circles indicate other FP/FOE positions. FP: fixation point; FOE: focus of expansion.

The monkeys were trained to look at a fixation point (FP) formed by a square pattern of two red vertical bars (0.17° wide) separated by a dark gap (0.17° wide), in a reaction time task (Figure 1(a)). At the FP onset, the monkeys had to push a lever beginning fixation (Figure 1(a)(A)). After 1000 ms the random dots appeared (Figure 1(a)(B)). Then, after 1000 ms, the expansion motion started (Figure 1(a)(C)), and after 1000 ms the dots inverted direction beginning the contraction motion (Figure 1(a)(D)). Trial duration was random and varied between 4000 and 6000 ms. The monkeys had to detect a change in FP orientation and release the lever within 500 ms (Figure 1(a)(E)). For each correct trial the monkey received a drop of juice or water as a reward. Eye position was monitored monocularly, with a resolution of 0.1°, by an optoelectronic system that uses the corneal reflection of an infrared light beam [26]. Eye position signals were sampled at 62.5/s. The fixation goodness was checked offline. Trials with eye position outside a 1° window around FP were not included in the data analysis.

In this experiment, we varied the position of the focus of expansion (FOE) and FP to determine the influence of the eye position and/or the spatial FOE position on the activity of PEc neurons. In the “retinotopic” condition, the FOE of the optic flow stimulus was presented in one of nine locations in a 3 × 3 grid at 15° distance each, while the monkey looked at the FP presented in the center of the screen (Figure 1(b)). In the “eye position” condition, we reversed the FP/FOE positions presenting the FOE in the center and the FP in one of the nine locations (Figure 1(c)). Taken together, these two conditions allowed us to obtain a set of data with identical retinal stimulation but different eye position. In the “angle of gaze” condition, we presented FOE and FP in the same position in each of the nine locations (Figure 1(d)).

### 2.3. Data Analysis

For each correct trial the firing rate was analyzed in the four stimulations (fixation, static dots, expansion, and contraction). The baseline activity was computed when the monkey looked at the FP with no visual stimuli presented on the screen (Figure 1(a)(A)). The spiking activity of the first 100 ms was discarded from the computation to avoid the effect of the saccade made to reach the FP. The aim of this analysis was to find the best model that described the pattern of activity. The onset and the end of each phase of neural activity were determined using a spline interpolation function having the baseline activity as threshold. This analysis also allowed quantifying the latency of each phase from the stimulus onset or from the end of the previous phase. All analyses were performed using Matlab (The MathWorks Inc.).

Once the phase latency and duration are determined, a univariate analysis of variance was performed on each parameter separately (mean phase duration, mean latency duration, and number of phases), in which condition (retinotopy, eye position, and angle of gaze), optic flow direction (expansion, contraction) and FOE/eye position (−15/−15; −15/0; −15/15; 0/−15; 0/0; 0/15; 15/−15; 15/0; 15/15) were the fixed factors. Effect sizes were calculated using partial eta squared (*η*p^2^), and means were considered significantly different at *p* < 0.05.

## 3. Results

We recorded the activity of 287 neurons in five hemispheres of three macaque monkeys. The statistical analysis performed after recordings showed that 152 neurons were significantly activated by at least one optic flow stimulus condition (one-way ANOVA, *p* < 0.05), while 135 cells were classified as nonvisual. Because of the very different pattern of activity, the results are illustrated according to the FOE/eye position in each condition.

**Figure 2:**
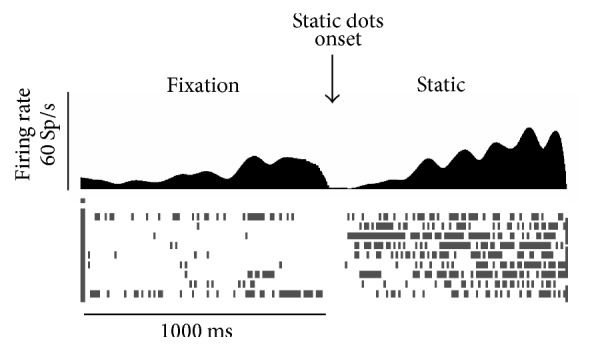
Pattern of activity of a PEc exemplary neuron during fixation and static dots presentation. On the top spike density plots (50 ms bin). On the bottom raster plot of firing rates, each vertical bar represents a single action potential. Spikes are aligned with the lever press. Data set: unit F232.

**Figure 3:**
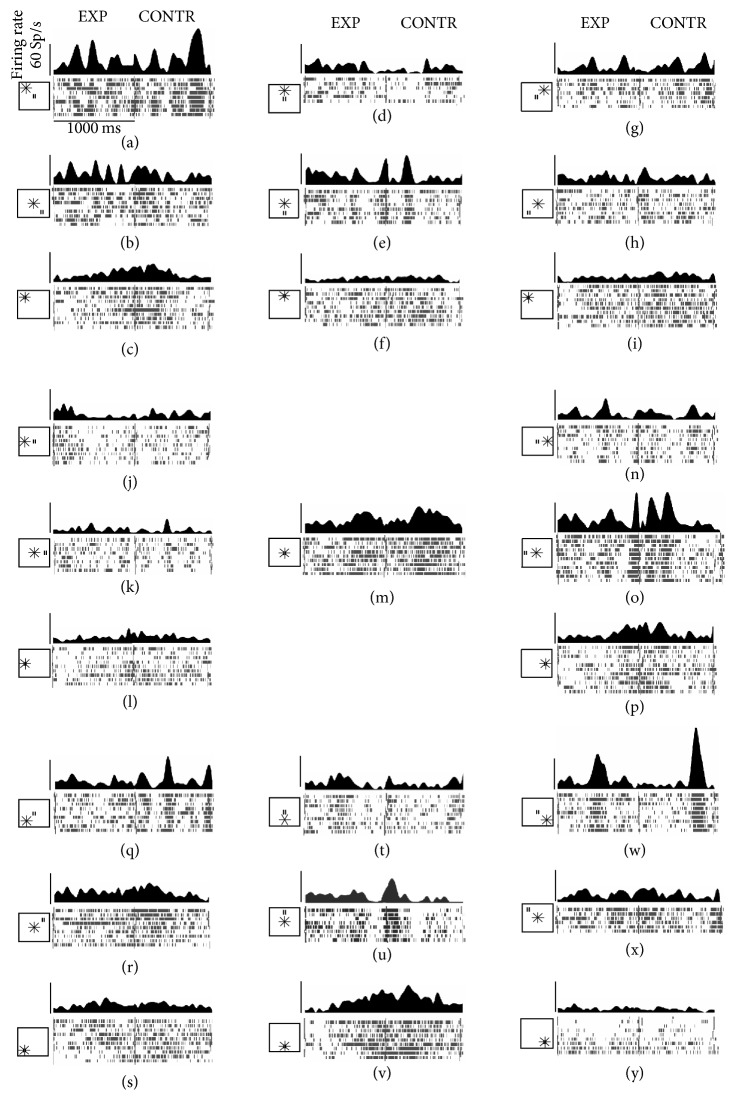
Pattern of activity of a PEc exemplary neuron: multiphasic and burst/pauses activity. On the top spike density plots (50 ms bin). On the bottom raster plot of firing rates, each vertical bar represents a single action potential. Spikes are aligned with the lever press. (a)–(y) Neural activity during expanding (EXP) and contracting (CONTR) radial optic flow stimuli, with nine different foci of expansion (FOE) and/or fixation. The (x)/(y) position of the FOE/FP is reported at the left of the raster plot. Data set: unit F232, the same cell as in Figure 2. Conventions as in Figure 1.

The phase determination analysis has been carried out in each visual neuron for each FOE and/or eye position for every condition resulting in a data set composed of 4060 different stimulations (44 conditions did not have a sufficient number of trials, so they were not considered for the analysis). Results showed that PEc neurons, in the majority of conditions (3059 stimulations, 75%), showed tonic activity for the entire period of optic flow stimulation. This set of conditions was not analyzed further. In the remaining comparisons, however (1001 stimulations, 25%), PEc neurons showed a very particular pattern of activity only during the optic flow stimulation, while no phase has been detected during the fixation or the static dots presentation (Figure 2). The most interesting finding is that such pattern was organized in excitatory phases that were interspersed with periods of inhibition or pauses. An example of such PEc neuronal pattern is shown in Figure 3, where the spike density and raster plots illustrate the phasic pattern in various conditions of an exemplary neuron. The plots are grouped according to the similarity of the conditions (the same retinotopy but different eye position and concentric FOE/FP). Considering the evaluation comparisons between conditions, the examples of phasic pattern of activity are visible in all comparisons; the strongest differences are visible in Figures 3(a)–3(c), Figures 3(g)–3(i), Figures 3(n)–3(p), Figures 3(t)–3(v), and Figures 3(w)–3(y). Clear examples of the burst/pause activity are visible in Figures 3(a), 3(e), 3(o), 3(q), 3(u), and 3(w). PEc neurons, in some case, also show a ramping-like activity (Figures 3(c), 3(m), and 3(v)). Regarding the described activity profile, 115 cells (115/152, 76%) showed phasic activity in specific conditions, while 37 cells (37/152, 24%) showed exclusively tonic activity.

**Figure 4:**
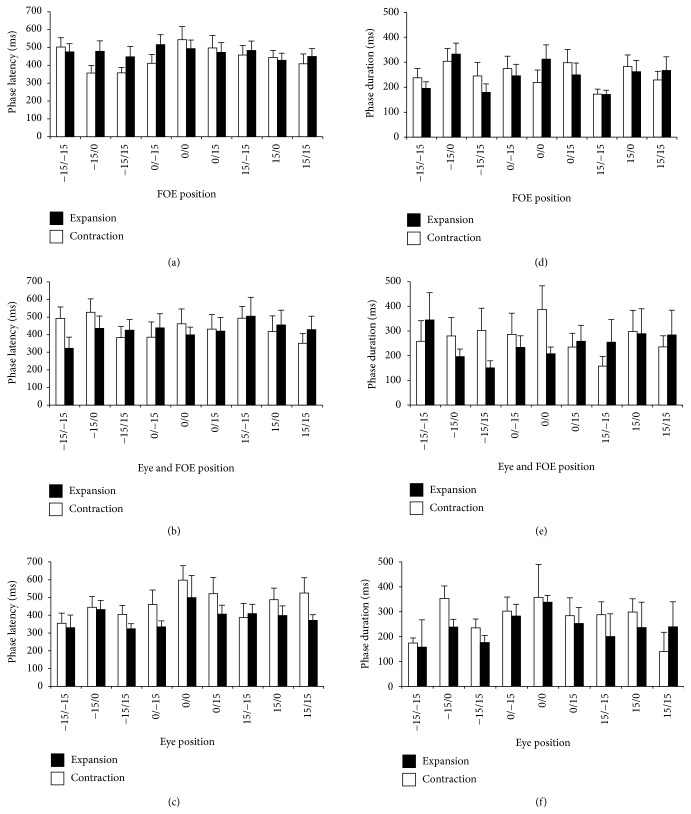
Mean latency differs across conditions and optic flow stimuli. Average values ± standard errors of phase latency and duration. Values are shown for expansion and contraction in the three conditions. (a) Phase latency in the retinotopic condition. (b) Phase latency in the eye position condition. (c) Phase latency in the angle of gaze condition. (d) Phase duration in the retinotopic condition. (e) Phase duration in the eye position condition. (f) Phase duration in the angle of gaze condition. Conventions as in Figure 1.

The phase onset and end were determined using a spline function having the baseline activity as threshold (see Methods). This analysis allowed determining the latencies, the phase duration, and the number of phases of each neuron in each condition. As visible in Figure 4, the mean latency is different across conditions and optic flow stimuli, being much higher in the retinotopic condition (Figure 4(a)) with respect to the angle of gaze (Figure 4(b)) and eye position (Figure 4(c)). The univariate ANOVA revealed a significant interaction effect between condition and optic flow direction (*F*_2,1244_ = 3.099; *p* = 0.045; np^2^ = 0.05). No significant difference has been found in the phase duration (Figures 4(d)–4(f)).

The analysis of the number of phases revealed a significant main effect of condition (*F*_2,1266_ = 48.9; *p* < 0.001; np^2^ = 0.72) with a mean value of 2.40 ± 0.04 for retinotopy, a mean value of 2.36 ± 0.06 for the angle of gaze, and a mean value of 3.12 ± 0.06 for the eye position. We also found a significant main effect of optic flow direction (*F*_1,1266_ = 78.16; *p* < 0.001; np^2^ = 0.58) with a mean value of 2.93 ± 0.04 for expansion and a mean value of 2.33 ± 0.04 for contraction, as well as a significant effect of interaction between condition and optic flow direction (*F*_2,1266_ = 58.12; *p* < 0.001; np^2^ = 0.84).

**Figure 5:**
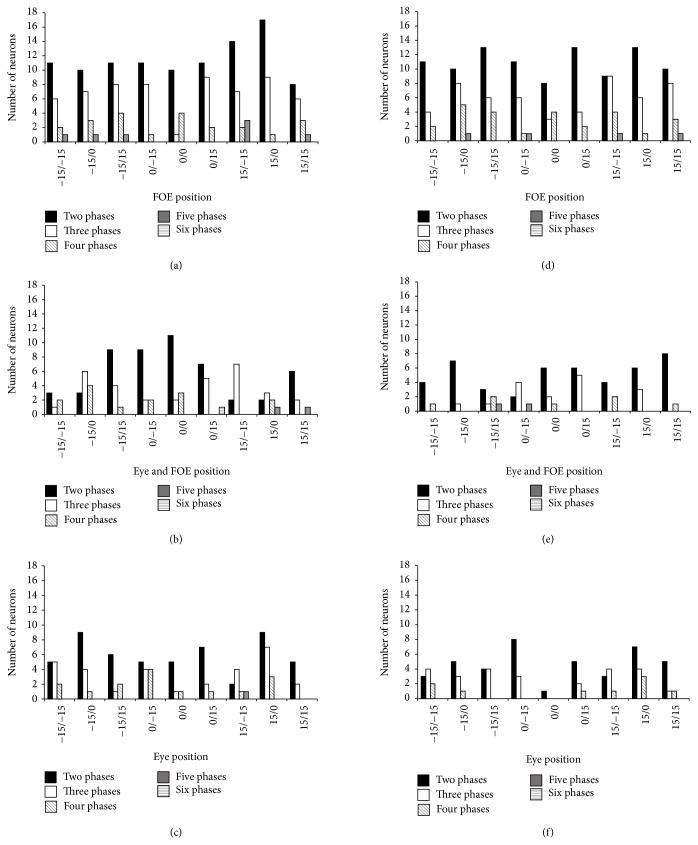
Number of phases recorded across conditions in PEc cells. Neurons are grouped by the number of phases to show their different phasic activity. Frequency distribution histograms indicating the number of PEc neurons that showed two or more phases during the entire period of stimulation. (a) Expansion stimulus in the retinotopic condition. (b) Expansion stimulus in the eye position condition. (c) Expansion stimulus in the angle of gaze condition. (d) Contraction stimulus in the retinotopic condition. (e) Contraction stimulus in the eye position condition. (f) Contraction stimulus in the angle of gaze condition. Conventions as in Figure 1.

Figure 5 shows the number of phases recorded across conditions in PEc cells. Neurons have been grouped by the number of phases to elucidate their different phasic activity. As visible, the majority of the neurons showed two phases during the optic flow stimulus presentation, but many neurons show three or more phases (see also the spike density and raster plots in Figure 3).

## 4. Discussion

In this study, we analyzed the pattern of firing activity of PEc optic flow visual neurons. We showed that many PEc neurons have a peculiar pattern of activity, which is related to a specific combination of FOE and/or eye position. An important characteristic of such pattern of activity is that excitatory phases are interspersed with inhibitory pauses. Further, in some case such phases ramped up or down during optic flow stimulation. This finding may shed some light on the cortical dynamics within area PEc and between PEc neurons and premotor/motor areas.

### 4.1. Origin of the Multiphasic Pattern

The most interesting characteristic of PEc phasic pattern is that the phase duration and the phase latency (or inhibitory pause activity) change according to the FOE/eye position or to a specific combination of them. It could be proposed that the phasic pattern would arise from the functional characteristics of the visual receptive fields. As already reported, the majority of PEc visual neurons have very large receptive fields usually extending over 30° with a broad directional selectivity [4, 27], with some of them showing a foveal-sparing receptive field with a robust response to both inward and outward directions [1]. It could be possible that these large receptive fields possess a microstructure made by tiny inhibitory/excitatory bands, which could elicit the inhibitory/excitatory pattern. Although this hypothesis may be compelling, it does not explain why the multiphasic pattern differs for the same retinotopy at different eye position (cf. Figure 3). Taking into account this finding together with the lack of phases during fixation and static dots presentation, we conclude that the phasic pattern may primarily result from a combination of FOE and eye position.

### 4.2. Functionality of the Parietofrontal Network

PEc neurons are directly connected to motor neurons in both premotor and motor cortex [8–13]. This parietofrontal circuit is thus involved in spatial stimuli localization during self-motion and/or body movement programming [1] and in restoring the spatial map after gaze shift [7]. Our present findings of PEc multiphasic activity pattern, especially its inhibitory pauses, suggest hypotheses about the information processing within this parietomotor circuit. First, we wonder about the role of alternate excitatory/inhibitory activity in activating premotor/motor neurons during self-motion. Mountcastle and coworkers [28] hypothesized that the posterior parietal cortex plays an important role in the “internal command” for action. Recent studies had further extended this idea by suggesting that movement initiation, when self-triggered or prompted by visual cue, might be driven by positive feedback processing involving corticobasal ganglia connections [21, 22]. Sanger [29] postulated that a closed-loop network is ideal for amplifying responses through positive feedback signals. A confirmation of this theoretic framework is the evidence of closed anatomical loops involving cortex with the basal ganglia or the cerebellum [30–32]. For example, it has been shown in anesthetized monkeys that electrical stimulation of cerebellar nuclei evoked cortical field potentials in the superior parietal lobule, suggesting selective cerebello-thalamo-parietal loop [33]. It is thus critical to consider the role of each neural node within its related cortical circuit. In this context, specific neural functional features observed in a cortical area can give us information about its input and output to connected areas/structures. Taking into account the anatomical connectivity between parietal area PEc and motor areas, it is possible that the multiphasic pattern of activity of PEc optic flow neurons may prompt transient activation of the motor neurons. Another potential involvement of this phasic activity would include an inhibitory role of PEc optic flow neurons on motor target neurons. It is reasonable to hypothesize that the firing pauses could cause the disinhibition of microcircuits within premotor/motor neurons via the involvement of local interneurons. In the parietal area 5, Maimon and Assad [21] found that a subpopulation of neurons, the so-called “dip neurons”, ramped down their firing activity well before the self-initiated movement. Such neurons were recorded more laterally with respect to those of the present study, still in the same parietal region. It is reasonable to hypothesize that the firing pauses could cause the disinhibition of microcircuits within premotor neurons via the involvement of premotor interneurons. Within this framework, the multiphasic activity of PEc optic flow neurons may thus provide the onset for the activation/inhibition of the loop, which would eventually lead to motor responses, depending on the spatial position of the visual stimuli.

Parietomotor network organization and processing develop and mature during childhood and are still plastic during learning in adults. The functional organization of subcortical and cortical connections has been extensively studied in the past decades [34]. Eaton and coworkers [35] showed that in monkeys the firing rates of motor cortical neurons and muscle activity can be operantly reinforced through the delivery of rate-contingent stimulation of the ventral striatum. Thus, the repetitive activation of specific parietal cells with burst/pause activity might enhance the functionality of circuits involved in movement generation during self-motion perception.

As proposed by Lee and Assad [22] and Maimon and Assad [21], the generation of concerted movements would imply a systems-level threshold for movement initiation. In this context, future works need to be carried out for understanding the functional connectivity of the parietofrontal loop linking area PEc to premotor/motor areas. A potential experiment would require simultaneous recordings both in area PEc and motor/premotor areas with independent electrodes. The synaptic interactions could be identified by analyzing the average responses. The eventual degree of synchronization of firing activity between parietofrontal neurons would elucidate if the process of action generation during self-motion is mere feedforward processing across cortical areas or if it requires feedback signals from frontal areas to parietal neurons. According to the hypothesis of Sanger [29], these feedback signals might be used for the modulation of the neural responses. The analysis of the firing pattern of activity of the parietofrontal loop may elucidate the relationship between network nodes as well as the relationship between pattern stability and visual perception.

### 4.3. Functionality of the Phasic Activity

PEc optic flow neurons are involved in the spatial mapping of the visual field, informing premotor/motor neurons of the spatial location of salient stimuli. It is true that PEc optic flow neurons have very large receptive fields [4, 27], but what matters for these neurons is a precise combination between the FOE of the optic flow field and the position of the eye in the orbit. The activation of posterior parietal neurons has been studied in relation to goal directed eye and/or hand movements [36, 37]. Battaglia-Mayer and coworkers [36] showed that the activity of most PEc cells is related to the direction of movement and to the hand position, both being influenced by eye position. In this context, this phasic pattern seems to be a very efficient way to signal the spatial location of visual stimuli given that the same neuron is able to send different firing patterns, that is, different inputs to target areas, according to the FOE/eye position.

## 5. Conclusions

The present finding represents the first analysis of the pattern of activity of PEc optic flow neurons. We found that many PEc neurons show multiphasic activity, which is strictly related to the position of the eye, to the position of the FOE of the optic flow stimulus, or to a combination of the two. The most intriguing feature of PEc neural activity is that such multiphasic activity is comprised of excitatory phases interspersed with inhibitory pauses. Previous studies demonstrated that PEc optic flow neurons are involved in the spatial mapping of the visual field [1–4]. Thus, the phasic pattern would represent a very efficient way to signal the spatial location of visual stimuli during self-motion, given that each neuron produces various firing patterns, which causes different activation of the same target neurons.
